# Restrictive Diets in Patients with Fibromyalgia: State of the Art

**DOI:** 10.3390/biomedicines12030629

**Published:** 2024-03-12

**Authors:** Miriam Almirall, Marta Musté, Mayte Serrat, Rafael Touriño, Esther Espartal, Sara Marsal

**Affiliations:** Rheumatology Department, University Hospital Vall d’Hebron, 08035 Barcelona, Spain; marta.muste@vallhebron.cat (M.M.); mayte.serrat@vallhebron.cat (M.S.); rafael.tourino@vallhebron.cat (R.T.); esther.espartal@vallhebron.cat (E.E.); sara.marsal@vhir.org (S.M.)

**Keywords:** fibromyalgia, diet, food, nutrition

## Abstract

Around 20–30% of Fibromyalgia patients modify their dietary habits after diagnosis, including avoiding certain food groups such as cereals. In this systematic review, we used the PRISMA guidelines to select the main studies that have evaluated the effectiveness of restrictive diets, including elimination and vegetarian diets, in patients with Fibromyalgia. Data on vegetarian/vegan diets are more consistent than data on elimination diets due to higher quality and better results of the published studies. Although the results are favorable in most of the studies, their heterogenicity and the scarce and low quality of the evidence (small number of patients included, often non-randomized and uncontrolled studies and multiple confounding factors and biases) does not allow for a positive recommendation about these restrictive diets in Fibromyalgia patients. Several factors other than food restriction could influence the symptomatic and functional improvements observed after restrictive diets, such as the placebo effect, weight loss that often occurs, coexistence with gastrointestinal diseases and positive effects of unrestricted foods. We must advance more and improve in our knowledge of the effectiveness of restrictive diets and variables related to them before recommending them systematically to all patients with Fibromyalgia. Randomized, placebo-controlled trials with large sample sizes, longer follow-up periods and standardized outcome measures that explore predictors of dietary response are needed to better understand the relationship between Fibromyalgia and nutrition.

## 1. Introduction

Fibromyalgia (FM) is a chronic disease, with an unknown etiology, characterized by widespread musculoskeletal pain accompanied by fatigue, waking unrefreshed and cognitive and gastrointestinal disorders [1,2]. Treatment should aim to improve health-related quality of life and often requires a multidisciplinary approach, with a combination of non-pharmacological and pharmacological measures [3]. No treatment has demonstrated high efficacy in improving the main symptoms of the disease [3].

The low efficacy of available treatments could be one of the factors that determines that around 20–30% of patients with FM modify their dietary habits after diagnosis, including the avoidance of certain food groups [4,5]. According to different studies, patients with FM consume fewer cereals, dairy products, fruit and fish than healthy controls [5,6]. The lower consumption of different food groups could have a negative effect on their physical and mental health [7]. 

Lopez-Rodriguez et al. [5] also assessed dietary restriction using the revised Restraint Scale. They observed that 43% of FM patients had dietary restrictions, with a greater avoidance of lactose, cereals, vegetables, and caffeine than healthy controls [5].

In the context of the relationship between irritable bowel syndrome, or IBS, and FM [8], an increased intestinal permeability compared to healthy controls [9,10], and previous literature on the effectiveness of different types of restrictive diets in FM patients [11,12,13,14,15,16,17,18,19,20,21,22,23,24,25,26,27,28] could also influence their modification of dietary habits and their avoidance of food groups.

In this paper, we review and analyze the main studies that have evaluated the effectiveness of restrictive diets in patients with FM to shed more light on this matter and to contribute to their better nutrition, avoiding certain restrictive diets that are not supported on quality scientific evidence, which may have a negative impact on their health.

This review focuses on the effect of restrictive diets that suppress the consumption of food groups, including both elimination and vegetarian/vegan diets. The elimination diets evaluated are the gluten-free diet (GFD), low fermentable oligo-di-monosaccharides and polyols (FODMAP) diet, low excitotoxins diet and hypoallergenic elimination diet.

## 2. Material and Methods

### 2.1. Design

This systematic review has been carried out following the recommendations of the preferred reporting items for systematic reviews and meta-analysis (PRISMA) guidelines [29].

### 2.2. Eligibility Criteria

Studies that recruited people with FM (ACR criteria or medical diagnosis) and examined the effect of restrictive diets (elimination and vegetarian/vegan) were selected for inclusion in this systematic review. Studies controlled with placebo or other diets were preferred, but for each type of diet, if there were no more than two controlled trials, uncontrolled studies were included.

### 2.3. Search Strategy

The strategy was to search for the terms “diet” and “fibromyalgia” in PubMed, refusing to evaluate full-text articles that were not included in the eligibility criteria.

An additional hand search was performed in the bibliographic references of other systematic reviews, including two new studies not found in the databases.

### 2.4. Data Collection

Data collection consisted of carefully screening the titles and abstracts of articles identified in the search strategy and carefully reading the screened full-text articles for eligibility to exclude articles that did not meet predetermined criteria. No specific data systems were used.

## 3. Results

A total of 181 articles were identified through database searching (PubMed) and 2 articles were identified through other sources (hand search in bibliographic references). Of the 183 selected articles, 149 were excluded for the following reasons: not related to dietary interventions and/or referred to diseases other than Fibromyalgia (*n* = 105), non-restrictive diets (*n* = 6), hypocaloric diets (*n* = 4), dietary supplements (*n* = 26), animal studies (*n* = 2), protocols (*n* = 4) and guides and recommendations (*n* = 2). 

The full texts of the remaining 34 articles were analyzed, among which 16 were excluded for the following reasons: reviews (*n* = 12) and uncontrolled studies on a diet that has more than two controlled trials (*n* = 4). 

**Figure 1:**
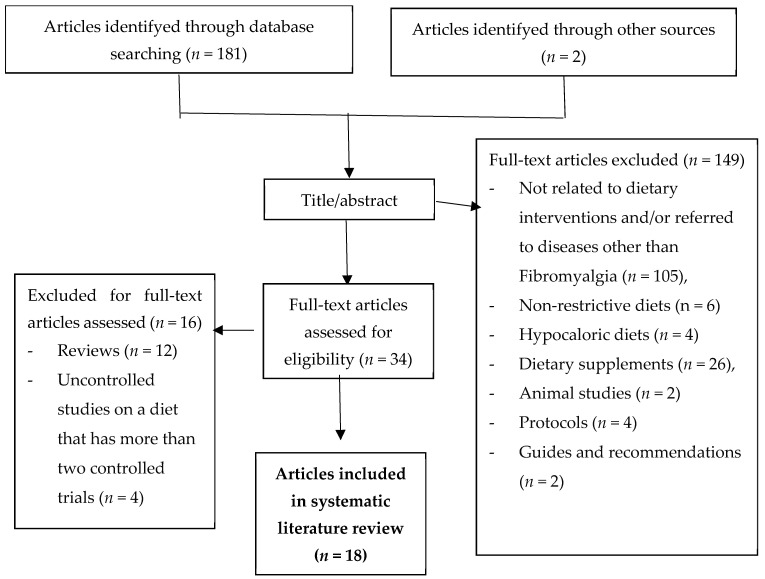
Flow chart of the systematic literature review.

A total of 18 articles were included in this systematic literature review (Figure 1).

### 3.1. Gluten-Free Diet

Patients with FM consume fewer cereals than healthy controls [5,6], although this dietary restriction is not well supported by the inconclusive results on the effect of a GFD in published studies [11,12,13,14,15,16,17,18].

In five studies, carried out in patients with FM and gluten-related disorders or intraepithelial lymphocytosis, symptomatic and functional improvement was observed after following a GFD [11,12,13,14,15]. In four of these studies, the duration of the GFD was at least 6 months [11,12,13,15].

In the first study by Rodrigo et al., seven patients diagnosed with FM and celiac disease (CD) followed a GFD for one year with a significant improvement in all the outcome measures evaluated, with a decrease of 51 to 60% in the Tender Points test (TPs), Fibromyalgia Impact Questionnaire (FIQ), Health Assessment Questionnaire (HAQ) and Visual Analogue Scales (VAS) for gastrointestinal complaints, pain and tiredness, and an increase of 48 to 60% in Short Form Health Survey (SF-36) [11]. In the subsequent study by Rodrigo et al. they evaluated 97 more patients, this time with FM and IBS, of which 58 had intraepithelial lymphocytosis (Marsh stage 1). The patients followed a GFD for one year and all outcome measures improved, discretely but significantly, in the group of patients with intraepithelial lymphocytosis, but not in the rest, with a decrease of 26 to 29% in the TPs, FIQ, HAQ and VAS scales (gastrointestinal complaints, pain and fatigue), accompanied by an increase of 27% in the SF-36 physical and mental component scores in Marsh stage 1 group [12]. Isasi et al. described the evolution of 20 patients with Fibromyalgia and intraepithelial lymphocytosis who improved after undergoing a GFD for a mean time of 16.4 months. Of the 20 patients, all presented symptomatic improvement but 15 presented the disappearance of generalized pain, 15 returned to work or normal life and 3 patients who were previously treated with opioids said that treatment was suspended. In this same article, Isasi reports that of his series, containing 246 patients with FM who underwent a GFD, 90 showed clinical improvement (36.6%), without providing more details in this regard [13].

Schinocca et al. also observed symptomatic and functional improvements (TP, FIQ, Widespread Pain Index (WPI) and Symptom Severity Scale (SS)) in 30 patients with FM and self-diagnosed non-celiac gluten sensitivity (NCGS) after following an 8 week GFD, with a significant decrease in the TPs (18 ± 0 to 12 ± 0; *p* < 0.02), FIQ (88.6 ± 3.3 to 67.5 ± 6.4; *p* < 0.001), WPI (16.3 ± 3.8 to 10.5 ± 3.5; *p* < 0.02) and SS (11.3 ± 0.8 to 7.2 ± 1.2; *p* < 0.0001) [14].

Slim et al. evaluated 75 patients with FM and NCGS symptoms. Although there were no differences in intestinal or extraintestinal symptoms, after following a hypocaloric diet or a GFD for 6 months, slight improvements were observed after both diets in number of gluten sensitivity symptoms (−2.13 ± 0.37 vs. −2.14 ± 0.4), body mass index (−1.12 ± 0.2 vs. −0.84 ± 0.2), FIQ (−8.58 ± 2.6 vs. −9.26 ± 2.8), Pittsburgh Sleep Quality Index (PSQI) (−1.1 ± 0.6 vs. −7.8 ± 0.62), Beck Depression Inventory-II (−3.06 ± 1.4 vs. −3.01 ± 1.5), Brief Pain Inventory-Interference (BPI-I) (−0.88 ± 0.29 vs. −0.48 ± 0.31) and Brief Pain Inventory-Severity (BPI-S) (−0.66 ± 0.25 vs. −0.67 ± 0.27) [15]. These results suggest that the weight loss that is often associated with restrictive diets could influence the improvements obtained.

Improvements in symptoms and functionality were detected after following a GFD in some, but not all cases in which FM presents comorbidly with gluten-related disorders.

Tovoli et al. retrospectively observed in a group of 13 patients with CD who also met FM criteria that their musculoskeletal symptoms did not improve after following a GFD [16].

Another study by Bruzzese et al., carried out in 20 women with FM and no history of IBS or other gastrointestinal diseases, evaluated the effectiveness of a therapeutic strategy based on a 6 month GFD, 3 month non-restricted gluten-containing diet and new rechallenge of a GFD for another 6 months. After 6 months of a GFD, a statistically significant reduction was observed for the WPI (10.3 ± 1.8 vs. 7.7 ± 1.4; *p* < 0.0001) and the SS scale (6.4 ± 1.8 vs. 4.15 ± 1.6; *p* = 0.0002). The D percentage reduction in the WPI after 6 months of a GFD was −24 ± 9%, while for the SS scale, it was −36 ± 21%. The following reintroduction of a gluten-containing diet brought about a statistically significant rise in the absolute SS scale and WPI, as well as a D modification in the WPI (21 ± 13%) and the SS scale (74 ± 90%). The rechallenge of the GFD showed a significant improvement in absolute and D WPI (−24 ± 7%) and SS (−36 ± 11%). Unlike other studies, no modifications to the body mass index were found [17]. 

Following these results obtained in mostly uncontrolled studies with very limited number of patients [11,12,13,14,15,16,17], our group carried out a study whose objectives were to establish the prevalence of NCGS, according to the Salerno Experts’ Criteria that included a placebo-controlled gluten challenge, in a cohort of FM patients and to evaluate their clinical response to a 6 week GFD [18]. In our cohort of 142 FM patients, the prevalence of NCGS was 5.6%, similar to that estimated in the general population (0.6 to 6%), and only 21.8% responded to the GFD, mainly at the expense of an improvement in intestinal symptoms. The presence of diarrhea and intraepithelial lymphocytosis and lower levels of anxiety were the predictive factors of GFD response. Based on these results, we concluded that GFD cannot be systematically recommended to all patients with FM, although it could be evaluated in those with diarrhea or intraepithelial lymphocytosis to assess improvements in their intestinal symptoms. In this study, we also evidenced the importance of the placebo effect in diet response, given that 74.2% of responders to GFD did not fulfill the NCGS diagnostic criteria because they did not discriminate between gluten and placebo in the placebo-controlled gluten challenge [18].

With the hypotheses that expectations for symptom reduction can improve the results obtained from any intervention, including diet, and that these expectations can be positively influenced by the given information regarding the placebos, a trial protocol that investigated this topic has been published. Patients with FM were randomly assigned into four groups: (a) gluten-free porridge + neutral open placebo (OP) instructions; (b) gluten-free porridge + positive OP instructions; (c) gluten-containing porridge + neutral OP instructions; and (d) gluten-containing porridge + positive OP instructions. Pain intensity, the occurrence of indigestion, functional capacity, treatment expectation and different pain-related and inflammation-related blood parameters were evaluated [30].

Besides the placebo effect, it is also of interest to study whether components of wheat other than gluten, such as amylase/trypsin inhibitors, wheat agglutinins or FODMAPs, could contribute to the improvements observed in patients with FM after following a DSG.

Finally, Silva et al. evaluated, in only 46 FM patients, the effectiveness of a 3 month anti-inflammatory diet, low in FODMAPs in the first month in a two-arm randomized controlled trial. The anti-inflammatory diet was rich in antioxidants, omega-3 and fiber and excluded gluten, dairy, added sugar and ultra-processed foods. The intervention group (*n* = 22) followed the anti-inflammatory and restrictive diet and the control group (*n* = 24) followed healthy non-restrictive diet recommendations. After intervention, there was an improvement in the intervention group compared to the control group in scores of FIQ (−19.9 ± 18.8 vs. −2.2 ± 16.1; *p* = 0.001), VAS-pain (−2.3 ± 2.5 vs. −0.04 ± 2.1; *p* = 0.002), BPI (−3.8 ± 4.1 vs. −1.1 ± 2.6; *p* = 0.011), FSS (−1.1 ± 1.2 vs. −0.5 ± 1.0; *p* = 0.042), VAS-GI (−2.0 ± 0.9 vs. −0.9 ± 1.3; *p* = 0.002), PSQI (−3.5 ± 4.6 vs. −1.2 ± 2.6; *p* = 0.048) and SF36 (10.2 ± 11.2 vs. 3.6 ± 10.4; *p* = 0.045) [19]. Symptomatic and functional improvements could be attributed to the anti-inflammatory diet properties, to dietary restrictions, to the combination of both or to other non-analyzed factors. 

In summary, the studies that show symptomatic and functional improvements after following a GFD are studies with small number of patients, often with gluten-related disorders or with intraepithelial lymphocytosis, not placebo-controlled (only one controlled with another diet) and with biases such as weight loss [11,12,13,14,15,16,17,18]. 

Therefore, the current evidence on GFDs in patients with FM (Table 1) does not allow us to recommend this restrictive diet.

Randomized, placebo-controlled trials with a large number of patients that explore the predictors of response are needed to analyze the true effectiveness of a GFD and the variables related to them in patients with FM, with special focus on weight loss and the presence of intraepithelial lymphocytosis and comorbid gastrointestinal diseases.

### 3.2. Low-FODMAP Diet

FODMAPs are short-chain carbohydrates and polyols that are fermentable and poorly absorbed by the small intestine, including fructose, lactose, fructans, fructooligosaccharides galactooligosaccharides, sorbitol and manitol [31].

There is significant evidence that the follow-up of a low-FODMAP diet might be a feasible first-line therapeutic strategy to reduce stomach discomfort, pain, bloating, and quality of life for patients with IBS [32] and that around 70% of FM patients also report IBS symptoms [33].

Apart from the study about anti-inflammatory and restrictive diets by Silva et al. [19], an uncontrolled study with only 38 FM patients examined the effectiveness of a 4 week low-FODMAP diet. A total of 88% of patients had gastrointestinal disorders and 60% had food intolerances, which could represent a significant selection bias. The patients significantly improved their functionality measured by FIQ and their intestinal and extra-intestinal symptoms (pain, fatigue, memory, sleep, headache and depression) (*p* < 0.01) [20]. Significant weight loss was also observed after the low-FODMAP diet.

The small number of patients included, absence of a control group and presence of multiple confounding factors and biases (such as gastrointestinal comorbidity and weight loss) do not allow the extraction of solid conclusions from this research. 

### 3.3. Low-Excitotoxins Diets

Monosodium glutamate and aspartame are excitotoxins that act as excitatory neurotransmitters and could cause neurotoxicity and the worsening of central and peripheral sensitization if used in excess [21].

Two case series with few patients [21,22] and two studies with a higher number of participants and better designs [23,24] evaluated the effectiveness of glutamate and/or aspartame elimination diets in patients with FM. These studies have shown inconsistent and contradictory results that do not allow for recommendations about these restrictive diets in patients with FM.

The first two studies analyzed four and two FM patients, respectively, and a complete or almost complete resolution of the symptoms after following a glutamate and/or aspartame elimination diet was shown. The diet duration was not specified [21,22].

In Horton et al.’s study, 26 patients with FM and IBS who had previously responded to a four-week excitotoxin-free diet (>30% improvement in symptoms) performed a two-week double-blind placebo-controlled crossover challenge with glutamate or placebo [23]. Patients worsened with the glutamate challenge with significant differences in FIQ (48 ± 22 vs. 36 ± 19; *p* < 0.03) and non-significant differences in pain VAS score [23].

In the last study, by Vellisca et al., a total of 72 patients with FM were randomized to the dietary elimination of monosodium glutamate and aspartame (*n* = 36) or waiting list (*n* = 36). Patients were requested to rate their pain using a seven-point scale. Comparisons between both groups showed no significant differences in pain referred during the baseline or three months after the elimination of excitotoxins [24].

### 3.4. Hypoallergenic Elimination Diet

Lamb et al. compared a standard American diet with a phytonutrient-rich hypoallergenic elimination diet in only eight women with FM. The hypoallergenic diet eliminated simple sugars, artificial colorants and sweeteners, caffeinated drinks, gluten-containing cereals, eggs, dairy products and foods high in arachidonic acid.

During the first 4 weeks, the participants consumed the American diet, and during the second 4 weeks, they were divided into two groups, namely, the hypoallergenic elimination diet and the American diet. Both programs (American and elimination diet) showed trends toward lower mean FIQ total score, Medical Symptoms Questionnaire (MSQ) total score, FibroQuest total score, FIQ stiffness score and FibroQuest headaches score. The elimination diet resulted in a significant decrease in the FIQ pain score compared to the standard American diet (−0.58 vs. 0.44; *p* < 0.05) [25].

Apart from the results obtained, this study has important limitations, such as the small number of patients included, absence of a placebo control group or the fact that we do not know if the limited benefits of the diet are due to restrictions or to the phytonutrients provided [25].

### 3.5. Vegetarian/Vegan Diets

All vegetarian/vegan diets are based on foods that come from plants with few or no ingredients that come from animals. This includes vegetables, fruits, nuts, seeds and cereals and excludes meat and fish. There are different subtypes; lacto-ovo-vegetarian includes dairy products and eggs, lacto-vegetarian excludes only eggs, and vegan or pure vegetarian excludes all animal-derived ingredients [34].

Vegetarian diets have been associated with improvements in biomarkers related to oxidative stress and inflammation [35], which are the mechanisms involved in the complex pathophysiology of FM [36].

In FM, data on vegetarian/vegan diets (Table 2) are more consistent than data on elimination diets due to higher quality and better results of the published studies. In this review, we focus on three clinical trials that were controlled with non-restrictive diets [26,27,28]. All included few patients, less than 35, and none had a double-blind design.

Hänninen et al. evaluated the efficacy of a 3 month uncooked vegan diet called the living food diet that consists of berries, fruits, vegetables and roots, nuts, germinated seeds and sprouts, i.e., rich sources of carotenoids and vitamins C and E. A total of 33 patients with FM were divided into the intervention group, with the living food diet, and the control group, with an omnivorous diet. They did not specify the number of patients assigned to each group. Patients in the intervention group showed a significant improvement in VAS-pain (−0.38; *p* = 0.003), stiffness (*p* = 0.001) and self-experienced health compared to the control group. They did not report whether there was weight loss [26].

Kaartinen et al. also evaluated the efficacy of the 3 month living food diet in 18 FM patients compared to an omnivorous diet control group (*n* = 15). The results revealed significant improvements in VAS-pain (*p* = 0.005), joint stiffness (*p* = 0.001), quality of sleep (*p* = 0.0001), HAQ (*p* = 0.031), general health questionnaire (GHQ) (*p* = 0.021) and a rheumatologist’s own questionnaire (*p* = 0.038) in intervention group. The majority of patients were overweight to some extent at the beginning of the study, and shifting to a vegan food diet caused a significant reduction in body mass index (*p* = 0.0001) [27].

Finally, in Martinez-Rodriguez et al.‘s trial, 21 FM women were randomly divided into three groups of seven participants: A (stabilization exercises and lacto-vegetarian diet), B (placebo and lacto-vegetarian diet) and C (control). The duration of the intervention was 4 weeks, and pain (VAS) and body composition (bioimpedance) assessments were performed at the beginning and at the end. Groups A and B showed significant changes compared to group C (*p* < 0.01 and *p* < 0.05), although group A was the one that showed a reduction in pain (−3.7 ± 1.4) and fat mass (−2 ± 0.7), without changes in weight. Therefore, although the lacto-vegetarian diet group showed differences to the control group, the most effective strategy was the combination of vegetarian diet and exercise [28].

## 4. Discussion

In FM, the low effectiveness of treatments [3], relationship with IBS [8], increased intestinal permeability [9,10], and previous literature on the effectiveness of restrictive diets [11,12,13,14,15,16,17,18,19,20,21,22,23,24,25,26,27,28] could contribute to changing the dietary habits of patients [4,5] with the avoidance of food groups such as cereals or dairy products [5], which could negatively affect their physical and mental health.

We conducted this systematic literature review to analyze the main studies that have evaluated the effectiveness of restrictive, elimination and vegetarian/vegan diets in patients with FM.

The studies that show symptomatic and functional improvements after following a GFD are studies with a small number of patients, often with gluten-related disorders or with intraepithelial lymphocytosis, not placebo-controlled (only one controlled with another diet) and with biases such as weight loss [11,12,13,14,15,17]. 

Weight loss in obese patientswith FM after following a hypocaloric diet has been associated with significant improvements in quality of life, depression, sleep quality and tender point counts [37]. Therefore, we do not know if the improvements observed after restrictive diets are mainly due to weight loss.

In studies with a greater number of patients, specifically 142 patients, less than 25% responded to a GFD and almost 75% of responders did not discriminate between gluten and placebo, suggesting other mechanisms involved in the response to diet, such as a placebo effect or components of wheat other than gluten [18].

The randomized, controlled trial that will investigate whether positive information regarding the placebos will improve expectations and reduce symptoms after administering gluten-free or gluten-containing porridge will shed further light on the influence of the placebo effect on the response to some diets [30].

Therefore, the current evidence on GFDs does not allow us to systematically recommend that this restrictive diet be followed by many FM patients.

In a low-FODMAPs study, the small number of patients included, absence of a control group and presence of multiple confounding factors and biases (such as gastrointestinal comorbidity and weight loss) [20] do not allow the extraction of solid conclusions from this research.

Studies regarding low-excitotoxins diets, with contradictory and inconsistent results [21,22,23,24], and about the hypoallergenic elimination diet, with important limitations (small sample size, absence of placebo control group and biases such as adding phytonutrients) [25], also do not allow us to recommend these restrictive diets.

Although data on vegetarian/vegan diets are more consistent due to the higher quality of the three clinical trials controlled with an omnivorous diet or placebo [26,27,28], its limitations (few patients, no double-blind designs, omitted or scarce data, few outcome measures and unanalyzed biases like weight loss) also do not allow its strongly recommendation in FM patients without carrying out further research. 

Finally, it should be noted that the benefit of plant-based diets could be more related to the consumption of foods with clear antioxidant and anti-inflammatory effects [38], such as fruits and vegetables, than to dietary restriction.

The two-arm randomized controlled trial by Silva et al. evaluated the effectiveness of an anti-inflammatory and restrictive diet [19]. Besides the small number of patients included, specifically 26, symptomatic and functional improvements could be attributed to the anti-inflammatory diet properties, to dietary restrictions, to the combination of both or to other non-analyzed factors. Therefore, we cannot conclude that the results of this study support the following of restrictive diets in patients with FM.

The main limitations of the published studies on the effects of restrictive diets in patients with FM were the heterogeneity in patient samples, designs and outcome measures, the low number of patients included, the sometimes scarce data, the short follow-up period, the studies designs often being non-randomized and uncontrolled and the multiple biases and unanalyzed confounding factors (weight loss, comorbidities and positive effects of unrestricted foods).

## 5. Conclusions

According to this review, data on plant-based diets, namely vegetarian and vegan diets [26,27,28], are more consistent than data on elimination diets, such as gluten-free [11,12,13,14,15,16,17,18], low-FODMAP [20], low-excitotoxins [21,22,23,24] and hypoallergenic diets [25], due to the higher quality and better results of the published studies.

Although the results are favorable in most of the studies, with symptomatic and functional improvements, their heterogenicity and the scarce and low quality of the evidence [11,12,13,14,15,19,20,21,22,23,25,26,27,28] do not allow for a positive recommendation about these restrictive diets in patients with FM.

Several factors other than food restriction could influence the symptomatic and functional improvements observed after following restrictive diets, such as the placebo effect, the weight loss that often occurs, coexistence with gastrointestinal diseases and positive effects of unrestricted foods [11,12,13,14,15,19,20,21,22,23,25,26,27,28].

Daily clinical practice should be based on quality scientific evidence. We must advance more and improve in our knowledge of the effects of different types of restrictive diets and related factors in patients with Fibromyalgia before recommending them systematically.

## 6. Future Directions

Randomized, placebo-controlled trials with larger sample sizes, longer follow-up periods and standardized outcome measures that explore the predictors of response are needed to analyze the true effectiveness of different diets and the variables related to them in patients with FM, with a special focus on weight loss and comorbid gastrointestinal diseases.

The predictors of response to different diets, the combined effectiveness of diets and other therapies, such as physical exercise, pain science education or cognitive behavioral therapy, and pathophysiological mechanisms involved in symptomatic and functional improvements and its relationship with alterations in gut microbiota and intestinal permeability should be evaluated and analyzed. 

Future studies should more rigorously delve deeper into the relationship between FM and nutrition, allowing us to better understand the gut–brain axis and the connection between gastrointestinal disorders and FM and to investigate more effective approaches to this complex disease.

## Figures and Tables

**Table 1:** Evidence of gluten-free diet in patients with Fibromyalgia.

Author, Year	GFD (*n*)	CG (*n*)	GRD and IL (*n*)	Duration	Weight Loss	Results
Rodrigo, 2013 [11]	7	No	CD (*n* = 7)	12 m	Nd	Symptomatic and functional improvement
Rodrigo, 2014 [12]	97	No	IL (*n* = 58)	12 m	Nd	Symptomatic and functional improvement in IL group
Isasi, 2014 [13]	20	No	IL (*n* = 20)	16.4 m	Nd	Improvement in all, remission in 15 patients
Schinocca, 2021 [14]	30	No	NCGS (*n* = 40)	8 w	Nd	Symptomatic and functional improvement
Slim, 2017 [15]	35	Hypocaloric diet (*n* = 40)	NCGS (*n* = 75)	24 w	Yes	Symptomatic and functional improvement with both diets (no significant differences)
Tovoli 2013 [16]	13	No	CD (*n* = 13)	Retrospective	Nd	Without improvement
Bruzzesse, 2023 [17]	20	No	No	6 m GFD 3 m URD 6 m GFD	No	Improvement of WPI and SS after GFD
Almirall, 2023 [18]	142	No	NCGS (*n* = 8) IL (*n* = 29)	6 w	Nd	21.8% responded to GFD due to intestinal improvement

GFD = gluten-free diet, *n* = number, CG = control group, GRD = gluten-related disorders, IL = intraepithelial lymphocytosis, m = months, w = weeks, CD = celiac disease, Nd = nondetermined, NCGS = non-celiac gluten sensitivity, URD = unrestricted diet, WPI = Widespread Pain Index, SS = Symptom Severity (SS) scale.

**Table 2:** Evidence relating to vegetarian/vegan diets in patients with Fibromyalgia.

Author, Year	IG (*n*)	CG (*n*)	Duration	Weight Loss	Results
Hänninen, 2020 [26]	Uncooked vegan diet (Unknown, TnP = 33)	Omnivorous diet (Unknown, TnP = 33)	3 months	Nd	Symptomatic improvement compared to CG
Kaartinen, 2000 [27]	Uncooked vegan diet (*n* = 18)	Omnivorous diet (*n* = 15)	3 months	Yes	Symptomatic and functional improvement compared to CG
Martinez-Rodriguez, 2018 [28]	Group A = exercise + lactovegetarian diet (*n* = 7) Group B = lactovegetarian diet (*n* = 7)	CG, Group C (*n* = 7)	4 weeks	No (reduction in fat mass)	Symptomatic improvement (pain) in group A (significant changes in groups A and B compared to group C)

IG = intervention group, *n* = number, CG = control group, TnP = total number of patients, Nd = nondetermined.
